# COVID-19: The Additional Sentence for the Incarcerated

**DOI:** 10.1089/heq.2020.0017

**Published:** 2020-09-30

**Authors:** Onyeka Otugo, Brooke Wages

**Affiliations:** ^1^ Department of Emergency Medicine, Brigham and Women's Hospital, Boston, Massachusetts, USA.; ^2^ Harvard Kennedy School of Government, Cambridge, Massachusetts, USA.

**Keywords:** incarcerated, COVID, disparity, prison, jail

## Abstract

Incarcerated people are one of the most vulnerable populations during the coronavirus pandemic. There are varying perspectives regarding how to address the health care barriers seen in this population. Some individuals and organizations advocate for a mandatory release of the incarcerated who are not deemed a risk to the general population, whereas others advocate for improving health care in jails and prisons. This article highlights the importance of addressing access to care issues, overcrowding, societal implications, and access to hygienics for the incarcerated during the coronavirus disease 2019 pandemic, and solutions forward.

## Introduction

The United States has the highest rate of incarceration in the world with one of the most expansive criminal justice systems in modern history. Approximately 2.3 million people are incarcerated, not taking into account those in juvenile detention, on home monitoring devices, or on probation.^1^ In all, there are ∼7 million individuals belonging to the United States criminal justice system. Incarceration disproportionately impacts minority populations in addition to those with mental health disorders.^2^ Compounding these issues are the unmet health care needs of this population. Those who are incarcerated have a disproportionate burden of diseases such as mental health illnesses, HIV/AIDS, substance use disorders, and chronic conditions such as diabetes and hypertension.^3,4^ This disproportionate burden results in poor health outcomes.^5^

On March 28, Patrick Jones, who was incarcerated in The Federal Correctional Complex in Louisiana, became the first known incarcerated person to die from COVID-19 after being diagnosed on March 19 (Refs.^6,7^). Coronavirus disease 2019 (COVID-19) does not discriminate against age, gender, creed, or incarceration status. Various prisons and jails have reported the spread of COVID-19 among their populations. It is not news that many prisons are overpopulated, creating the perfect breeding ground for infectious disease transmission leading to outbreaks. The lack of effective health care is a result of poorly resourced facilities and structural racism resulting in a disproportionate number of African Americans and Hispanics who are incarcerated.^1,8^ Simply stated, our health care system is failing this vulnerable population. With the growing number of those infected with COVID-19, these issues will only further be exaggerated. Incarceration presents significant disparities regarding health equity. Reentry after incarceration in the United States is a broken process, which is further intensified in the midst of a pandemic.

Communicable diseases pose serious risks to those in correctional facilities given the intimate confines, lack of access to adequate sanitation methods, and barriers to equitable health care.^9^ Diseases such as tuberculosis, hepatitis C, and HIV/AIDS are more common in correctional facilities.^4,5^ New York Governor Andrew Cuomo promoted an initiative to generate >100,000 gallons of hand sanitizer weekly produced by inmates. These individuals are producing the same hand sanitizer that is not made available to them for use in most prison facilities given their high alcohol content.^10^ In addition, it is not uncommon to find sinks that do not work or are not easily accessible and a lack of soap in prisons.^11^ Given that overcrowding and poor sanitation are vehicles to promote disease transmission, this population is left further exposed. An additional barrier to care is the copay requirement in certain state prisons and jails act as an additional barrier to care. Even though prisons provide health services, they can come with the cost of a copay.^12^ California and Illinois have taken steps forward by eliminating copay for the incarcerated, and others should follow.^13^

There are different schools of thought regarding what to do with the incarcerated population. Given the inadequate health care delivery system for the incarcerated, some view keeping the incarcerated in prisons and jails during the COVID-19 pandemic as an extension of their existing sentence. A proposed solution is the reduction of incarcerated people in jails and prisons to prevent the spread of COVID-19. Thousands of people have been released from correctional facilities in the past several weeks to mitigate the spread of COVID-19. Recently, the Committee for Public Counsel Services and the Massachusetts Association of Criminal Defense Lawyers filed an emergency petition that calls for the reduction of the number of incarcerated individuals through limiting those who will be taken into custody and releasing those who would be considered high risk for contracting COVID-19, those toward the end of their sentence, and those who do not pose a significant risk to the general population.^14^ Similarly, other states have filed similar emergency petitions to protect incarcerated people. However, will this intervention be the answer to prevent the pandemic from spreading?

A competing position is that the release of the incarcerated may further propagate the spread of COVID-19. A majority of the individuals in jail are pretrial detainees and are often placed in jail because they were unable to afford bail or were not granted bail. Since these individuals are in a preadjudication status, they are innocent as defined by the constitution. However, they often lack an effective re-entry plan in place if they are to be released.^15^ In addition, there is a greater burden of mental health and homelessness among the incarcerated, as well as higher rates of unemployment before and after incarceration.^16–18^ According to a report released by the U.S. Department of Justice Bureau of Justice Statistics released in 2017, *Indicators of Mental Health Problems Reported by Prisoners and Jail Inmates, 2011–2012*, 44% of jail inmates and 37% of prisoners self-reported that they were told by a medical professional that they had a mental health disorder.^19^ Other estimates of mental health disorders are much greater. Formerly incarcerated people are also 10 times more likely to be homeless in comparison with the general public according to a report by the Prison Policy Initiative.^20^ If these individuals are to be released, they would likely find themselves in shelters that are also overcrowded facilities with individuals living in close proximity to other individuals and further the spread of this pandemic. In addition, there is limited capacity of other nonprofit organizations for the homeless, such as soup kitchens, to meet the needs of this population.

Jail deincarceration is necessary but it must be balanced for the safety of the incarcerated and the public. It may be negligent to release thousands of people who will face hiring discrimination at the beginning of an economic downturn and in the middle of a pandemic. State and federal correctional facilities are reviewing records of incarcerated people to see whether they can be reclassified and possibly released or relocated to a less crowded unit or facility. A solution to this nuanced problem would be the voluntary release of incarcerated individuals from jails given the majority of individuals in jails are pretrial, with the exception that they are not deemed a threat to the public. In addition, ensuring that re-entering people have stable housing upon release is vital and some states are actually turning to hotels to meet this need. Advocating for voluntary jail release reflects the burden of homelessness, employment barriers and discrimination, and mental health in this population. Furthermore, it demonstrates understanding that placing re-entering people on the streets without employment opportunities or housing security will further worsen the current situation.

Nearly all in-person educational and personal development programming in jails and prisons are canceled.^21^ This not only removes healthy outlets for people on the inside but also removes opportunities to earn money working in nonessential parts of a prison, such as library facilities or participate in programs that can reduce a person's sentence. To mitigate the stress of increased isolation during social distancing, advocating for free texting, calling, and video chat services from prison telecommunications companies is essential. Given the mental health burden in prisons, it is necessary to take steps toward eliminating the charge associated with communication services. Currently, Edovo, a telecommunication and technology education company, has taken initiative by providing connection in the midst of social distancing by supplying free access to messaging and calling to the incarcerated.^22^

## Conclusion

To do no harm to this disenfranchised population, the first step is recognizing that there is a problem with health care in correctional facilities. It begins with accountability and continues with addressing the needs of this vulnerable population. Voluntary jail release offers a solution to supporting those who are incarcerated during this time. The solution also includes ensuring access to adequate health care, removing copays for medical care that often act as barriers to care, reducing overcrowding in facilities, as well as improving access to hygienic products. Furthermore, it is essential to address the mental health needs of this population which begins by understanding that social distancing does not equate to emotional distancing during this pandemic.

## Abbreviation Used

COVID-19: coronavirus disease 2019
